# Hemophagocytic lymphohistiocytosis and miliary tuberculosis in a previously healthy individual: a case report

**DOI:** 10.1186/s13256-020-02555-x

**Published:** 2020-11-11

**Authors:** Linn Hereide Trovik, Miriam Sandnes, Bjørn Blomberg, Gunhild Holmaas, Aymen Bushra Ahmed, Tor Henrik Anderson Tvedt, Olav Vintermyr, Håkon Reikvam

**Affiliations:** 1 Department of Medicine, Haukeland University Hospital, Bergen, Norway; 2 Department of Clinical Science, Institute of Clinical Science, University of Bergen, Bergen, Norway; 3 Department of Surgical Services, Haukeland University Hospital, Bergen, Norway; 4 Department of Pathology, Haukeland University Hospital, Bergen, Norway; 5 Departemnt of Medical Science, University of Bergen, Bergen, Norway

**Keywords:** Cytokines, Ferritin, Hemophagocytic lymphohistiocytosis, Infection tuberculosis

## Abstract

**Background:**

Hemophagocytic lymphohistiocytosis (HLH) is a rare heterogenous genetic or acquired hyperinflammatory syndrome associated with a high degree of morbidity and mortality. HLH has clinical manifestations related to abnormal prolonged activation of T lymphocytes and macrophages with an excess of proinflammatory cytokines. The main causes of secondary HLH are malignancies and infectious diseases.

**Case presentation:**

The patient was a 54-year-old man, originally from Eastern Africa, who had lived in Northern Europe for 30 years. Here we describe the clinical features, laboratory parameters, diagnostic workup, management and outcome data of a previously healthy 54-year-old man diagnosed with HLH secondary to tuberculosis. The patient was initially treated for a community-acquired pneumonia. He developed multiorgan failure with acute respiratory distress syndrome, hypertransaminasemia, and kidney and bone marrow dysfunction. The clinical course together with a simultaneous increase in serum ferritin raised the suspicion of HLH. The patient fulfilled seven out of eight diagnostic criteria for HLH. A thorough diagnostic workup with respect to HLH and a potential underlying disease was initiated. Cultivation of bronchoalveolar lavage fluid, stool and urine, and polymerase chain reaction of epithelioid cell granulomas in the bone marrow were all positive for *Mycobacterium tuberculosis*. He was treated for both HLH and tuberculosis, and he survived without any sequelae.

**Conclusions:**

We present one of few published cases of a patient who survived HLH triggered by miliary tuberculosis. The current case illustrates the need for awareness of these two diagnoses, and the timely initiation of specific and supportive treatment to reduce mortality.

## Introduction

Hemophagocytic lymphohistiocytosis (HLH) is an uncommon hematologic disorder characterized by an uncontrolled immune response with organ infiltration of lymphocytes and histiocytes, and organ damage caused by excessive production of pro-inflammatory cytokines [1–6]. HLH is categorized either as primary due to a genetic disorder, or as secondary due to an acquired condition. Secondary HLH can be triggered by neoplastic and non-neoplastic diseases [7]. Malignancies associated with secondary HLH are mainly various hematological malignancies such as leukemia or lymphoma, whereas autoimmune disorders and infectious diseases are the most common nonmalignant diseases associated with HLH [3, 8]. While active tuberculosis (TB) is rare in the Nordic countries, the World Health Organization estimates 10 million new TB cases globally causing 1.5 million deaths every year, thus making *Mycobacterium tuberculosis* the single most lethal infectious agent in the world [9, 10]. HLH due to TB is very uncommon, with only a few cases reported, mostly in immunocompromised patients [8, 11–13].

Here we report a case of HLH associated with miliary TB in an apparently immunocompetent healthy man. The case illustrates that the combination of an aggressive diagnostic approach, searching for a broad variety of disorders, combined with an early therapeutic intervention are crucial to securing a successful outcome.

## Case presentation

**Fig. 1:**
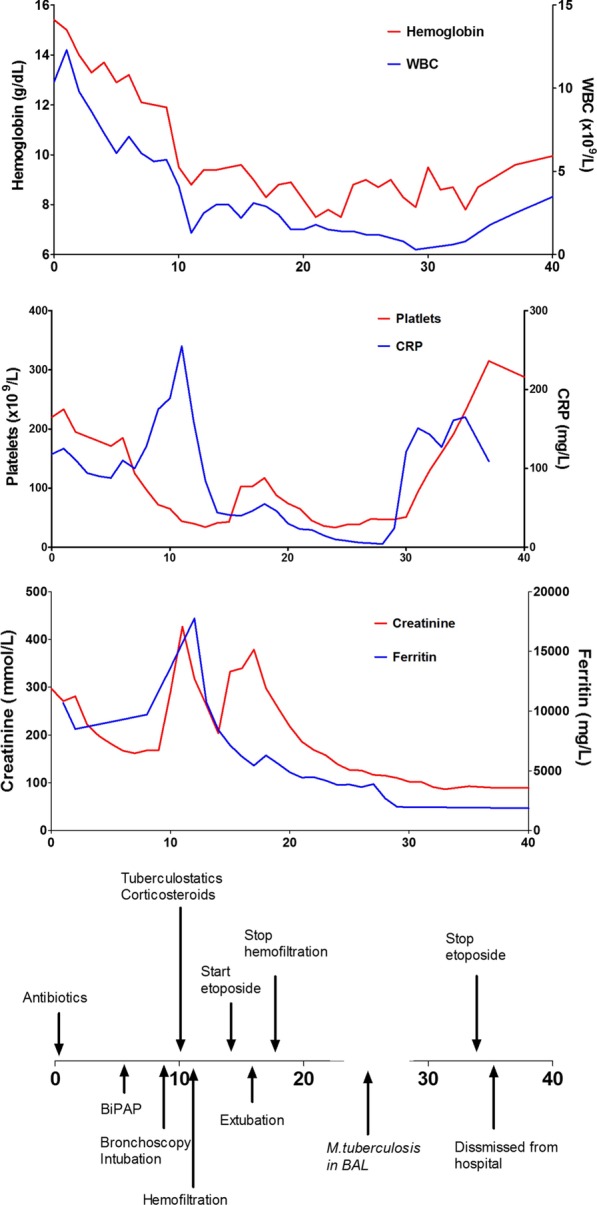
Development in biochemical parameters during the disease course. Day 0 is the first day of hospitalization, and the timing of various diagnostic and prognostic approaches are marked in the graph

**Fig. 2:**
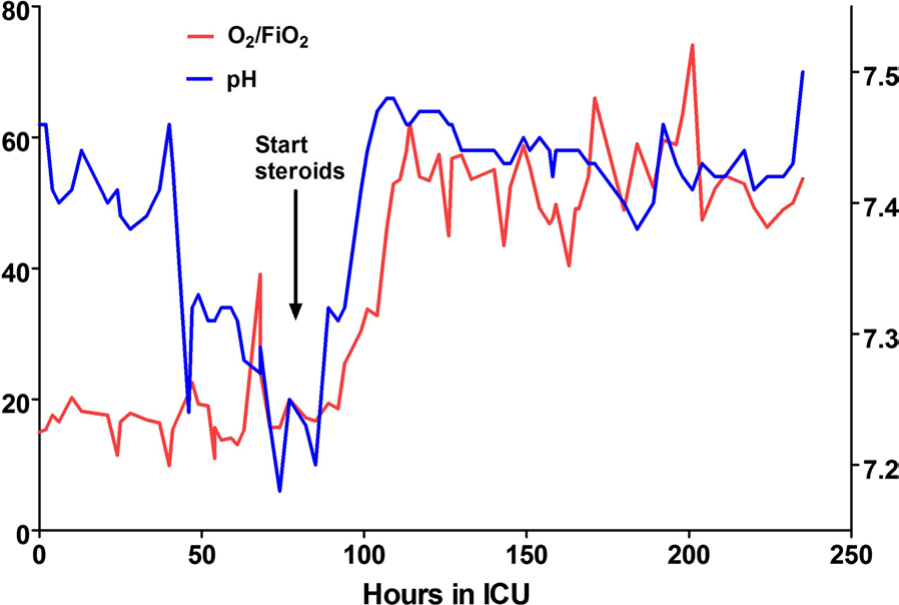
Oxygenation (paO_2_/F_i_O_2_ ratio) and ventilation (pH) during the intensive care unit (ICU) stay. Time is marked as hours after ICU attendance. The arrow marks the start time point for steroid treatment

**Fig. 3:**
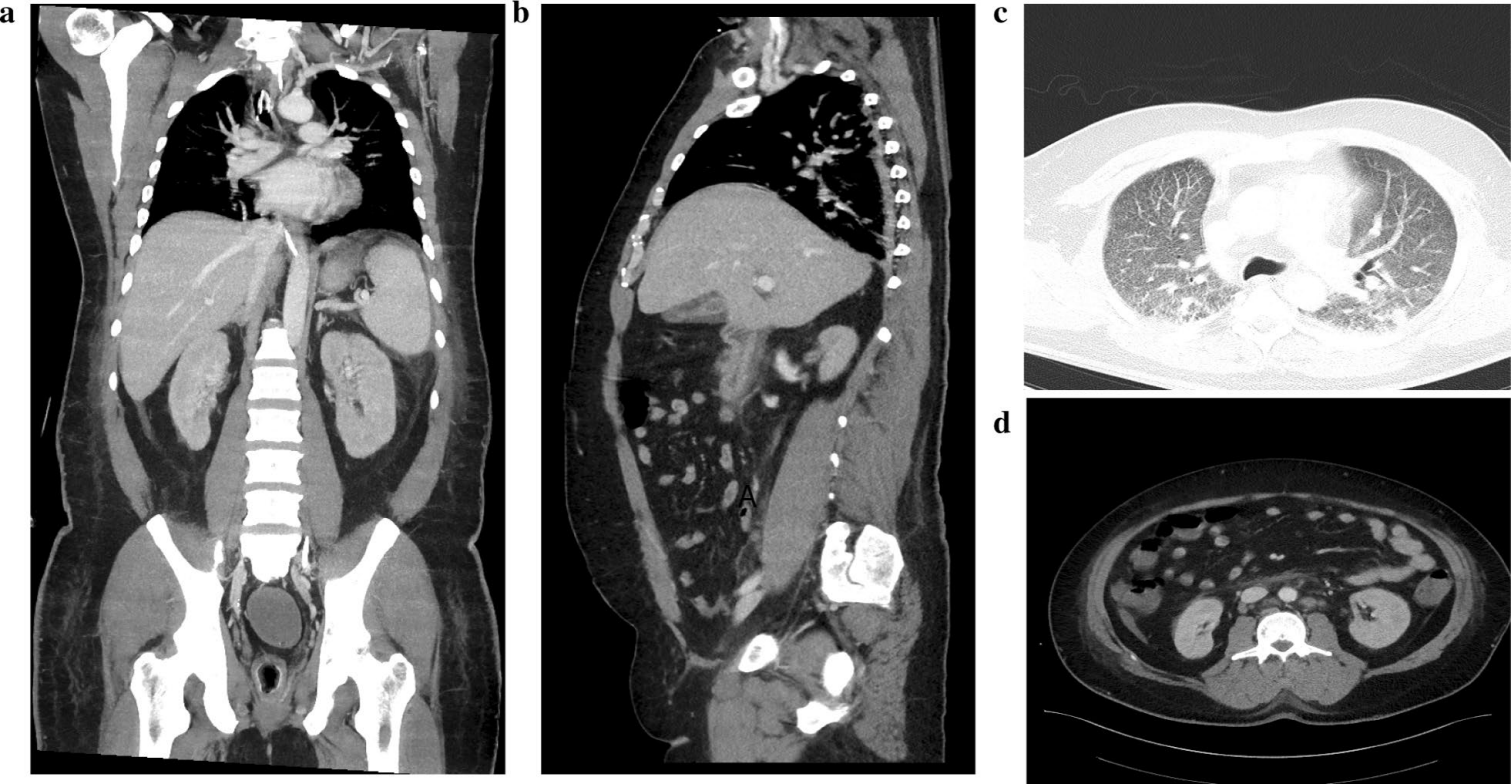
Radiological examination of the patient. Computed tomography scan images of the abdomen taken at admission demonstrating enlarged spleen and liver, lymphadenopathy in the abdomen, thickened wall of colon and bile duct, and atelectasis in the lung sections

The patient was a 54-year-old man, originally from Eastern Africa, who had lived in Northern Europe for 30 years. Apart from increased blood pressure, causing mild left ventricle hypertrophy, he was healthy and only taking antihypertensive medication. He contacted his family physician due to nausea and loose, yellowish stools. Due to dysuria and microhematuria, he had received treatment with orally administered mecillinam for a suspected urinary tract infection. As this treatment had no effect, he was given doxycycline on suspicion of respiratory tract infection due to dyspnea and fever. Eventually, he was admitted to the hospital after 10 days of nausea, anorexia, diarrhea, frequent micturition, dyspnea, persistent fever, increasing C-reactive protein (CRP) and elevated liver transaminases. He had no cough, night sweats or weight loss. On clinical examination on admission, he was alert and oriented, but hypotensive (blood pressure 102/58 mmHg) and hypoxic with SpO_2_ of 86% without supplemental oxygen. The responsible clinician described mild scleral jaundice, but no rashes or palpable adenopathy. Pulmonary auscultation revealed bilateral crackles. Laboratory test values showed hemoglobin level of 15.4 g/dL (reference 13.4–17.0), leukocyte count at 10.4 10^9^/L (ref 3.5–11.0) and thrombocytes at 220 10^9^/L (ref 145–348). CRP was elevated at 118 mg/L (ref < 5). Erythrocyte sedimentation rate was not taken at admission. He had acute kidney failure with creatinine at 297 µmol/L (ref 60–105), and elevated liver transaminases with alanine aminotransferase of 208 U/L (ref 10–70), alkaline phosphatase 191 U/L (ref 35–105), gamma-glutamyltransferase 337 U/L (ref 15–115) and bilirubin 26 µmol/L (ref < 20) (Fig. 1). He had severely reduced partial pressure of oxygen in arterial blood to the inspired oxygen ratio (paO_2_/FiO_2_) at 31.3 kPa (ref 55–65) (Fig. 2). Computed tomography (CT) scan revealed ground-glass opacities in both lungs, extensive unspecific changes in the colon, multiple enlarged para-aortic lymph nodes, fat tissue reaction in the posterior abdominal wall (Fig. 3), an enlarged liver at 19 cm in the midclavicular line with rounded margins, and a slightly enlarged spleen at 14 cm.

On admission, the clinical presentation was not clear-cut, and the attending clinician considered the possibility of viral or bacterial infections, including pneumonia (fever, dyspnea, auscultatory crackles), possibly due to atypical agents, as well as urinary tract infection, hepatitis, intra-abdominal infection, gastroenteritis with ensuing hemolytic uremic syndrome, and a variety of noninfectious diseases including renal failure due to dehydration, vasculitis, other systemic diseases, heart failure and thromboembolism. Broad-spectrum antibiotic (piperacillin-tazobactam) was started to cover for bacterial infection of unknown origin. No clinical improvement was observed the following week, and due to increasing respiratory failure, he was transferred to the nearest regional university hospital for bronchoscopy and further diagnostics. Piperacillin-tazobactam was given from admission and meropenem from day 7, with the addition of azithromycin from day 9 for a suspected bacterial etiology, but without any clinical or biochemical improvement. He developed increasing respiratory failure that required noninvasive ventilation from day 7, and progressive hypoxemia necessitated intubation at day 10. Continuous renal replacement therapy was initiated from day 11 due to increasing kidney failure as evidenced by decreasing diuresis, metabolic acidosis and increasing serum creatinine levels (Figs. 1, 2). Despite lung protective ventilatory support, the paO_2_/FiO_2_ rapidly deteriorated (Fig. 2). On day 11 after admission, the attending clinicians started treatment with high dosages of corticosteroids to cover for a possible autoimmune etiology.

At this point, the diagnostic assessment had not revealed the cause of the severe clinical course. Standard workup for infectious diseases with culturing of blood, urine and stools did not reveal the etiology. Serological tests for human immunodeficiency virus (HIV) and hepatitis A and B were all negative. Serology revealed prior but not active infection with Epstein–Barr virus (EBV) and cytomegalovirus (CMV). Since the patient had been back in his homeland 1 year prior to hospitalization, tropical infectious diseases with long incubation time were considered. With fever, pancytopenia and splenomegaly, he displayed the cardinal signs of visceral leishmaniasis, but microscopy of blood and bone marrow aspirate did not reveal visible *Leishmania* amastigotes, serology was negative and polymerase chain reaction (PCR) of blood could not detect *Leishmania* DNA. Serology for strongyloides was also negative. Considering the febrile illness with pulmonary opacities, respiratory failure and subacute deterioration of multiple organ systems, miliary TB was recognized as a possible diagnosis. Interferon gamma release essay (IGRA, QuantiFERON^®^) taken at admission was positive at a medium level at 1.98 IU/mL. Upon transfer to the regional hospital, a new IGRA test was taken with negative/inconclusive results, with a value of 0.21 IU/mL, and a repeat test showed a gray zone result of 0.39 IU/mL (reference values negative < 0.26, gray zone 0.26–0.43, low positive 0.44–0.69, medium positive 0.70–3.99, strong positive ≥ 4.00). No immunosuppressive treatment had been given at this point. Direct microscopy of bronchial fluid obtained by bronchoscopy did not show acid-fast bacilli, and PCR on the same specimen was negative for *Mycobacterium tuberculosis*.

However, these findings could not exclude TB infection. Therefore, the patient was given therapy with rifampicin and isoniazid intravenously, and pyrazinamide on nasogastric tube, against possible TB infection at the same time as steroids were started. Intravenous levofloxacin was added to cover for other potential bacterial infections. Although levofloxacin also has antimycobacterial properties, it is not part of standard first-line anti-TB treatment.

An exceptionally rapid improvement of the respiratory failure was seen after initiating treatment with high dosage of steroids and tuberculostatic drugs (Fig. 2). The kidney failure continued, however, and the bone marrow failure was worsening, with rapidly decreasing thrombocyte and white blood cell count (WBC) (Fig. 1). Since admission, the patient had persistent fever, increasing levels of serum ferritin to a maximum value of 17,000 µg/L (ref 34–300), splenomegaly and increasing pancytopenia, in addition to hypertriglyceridemia with serum triglycerides of 5.2 mmol/L (ref 0.45–2.60). Based on these clinical and laboratory findings, a diagnosis of HLH was suspected (Table 1). Two weeks after admission, blood tests were analyzed for soluble IL-2 receptor and natural killer (NK)-cell activity, and bone marrow biopsy was performed. An eliciting cause for HLH was sought. Tests for antinuclear antibody (ANA) and antineutrophil cytoplasmic antibody (ANCA) were both negative. Immunophenotyping of bone marrow showed no evidence of lymphoproliferative diseases with T- or B-cell clonality. Lymphadenopathy of the posterior abdominal wall and wall thickening of the colon seen on CT scan at admission disappeared after initiation of corticosteroid therapy, and biopsy was no longer possible.

**Table 1:** The diagnostic criteria for hemophagocytic lymphohistiocytosis with definitions

Findings	Definitions	Present
Fever	Peak temperature of > 38.5 °C for > 7 days	X
Splenomegaly	Spleen palpable > 3 cm below costal margin	X
Cytopenia	Involving > 2 cell lines; Hb < 9.0 g/dL, ANC < 1.0 × 10^9^/L, platelets < 100 × 10^9^/L	X
Hypertriglyceridemia or hypofibrinogenemia	Fasting triglycerides > 2.0 mmol/L or > 3 SD more than normal value for age or fibrinogen < 1.5 g/L or > 3 SD less than normal value for age	X
Hemophagocytosis	Hemophagocytosis demonstrated in biopsy samples of bone marrow, spleen or lymph nodes	X
Low or absent NK cell activity	Reduced NK cell activity as measured by standardized assay	
Hyperferritinemia	Serum ferritin > 500 ng/mL	X
Elevated soluble interleukin-2 (CD25) levels	CD25 level > 2400 U/mL	X

*Hb* hemoglobin, *ANC* absolute neutrophil count, *SD* standard deviation, *NK* natural killer

**Fig. 4:**
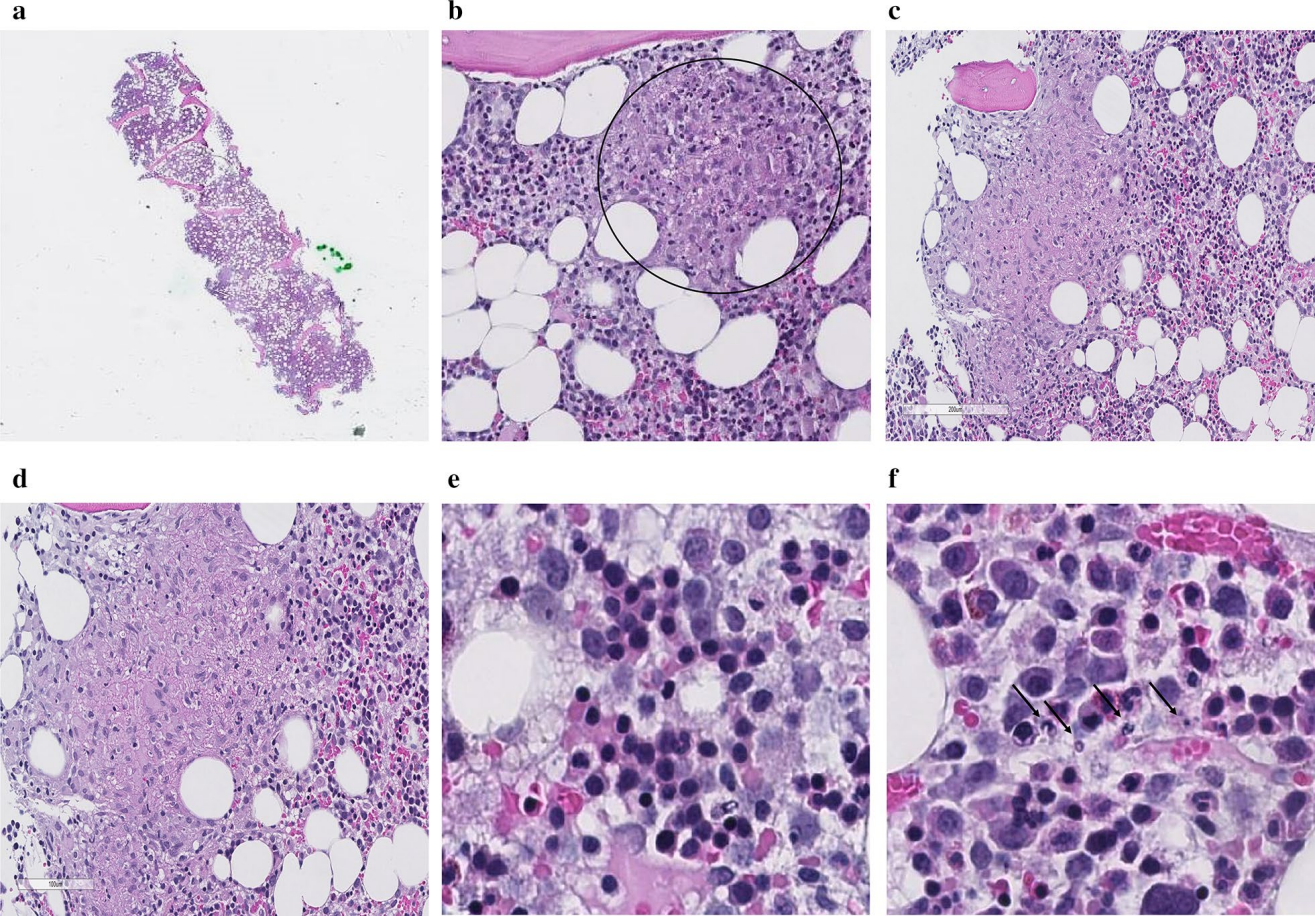
Histopathological features of the bone marrow. The figure demonstrates bone marrow findings for the patient with hematoxylin and eosin staining. **a** Overview of the bone marrow demonstrating hypercellular bone marrow with bone marrow cellularity of approximately 70%. **b** Encircled area displays groups of epithelioid cells consistent with granulomas. **c** Higher resolution demonstrating epitheliod cell granulomas located within the bone marrow. **d** Demonstrating granuloma with several giant cells present. **e** A significantly expanded erythropoiesis. **f** Several macrophages with intracytoplasmic residues of nuclear fragments consistent with hemophagocytosis (arrows)

At day 10, the patient fulfilled five out of eight diagnostic criteria for HLH (fever, splenomegaly, bi-cytopenia, hypertriglyceridemia and hyperferritinemia), and treatment with etoposide 75 mg/m^2^ was initiated according to the HLH-2004 protocol [14], although with 50% dose reduction due to cytopenia and hyperbilirubinemia, and without cyclosporine A (Fig. 1). Over the next days a rapid improvement of the kidney function, decrease in serum ferritin levels and improvement of the general condition were observed (Fig. 1). All anti-TB drugs were discontinued, as all tests for TB apart from the interferon-gamma release assay remained negative, and TB was considered less likely. Levofloxacin was continued. Three weeks after admission, however, culture of bronchial fluid, stool and urine revealed *M. tuberculosis* and was also positive on the antigen MPT64 assay, confirming the diagnosis of TB. Results from bone marrow biopsy later revealed an expanded erythropoiesis, increased number of histiocytic cells with phagocytosed lymphocytes and nuclear debris (hemophagocytosis) and granulomas that supported the diagnosis of HLH and TB, respectively (Fig. 4). The bone marrow was also later found to be positive for *M. tuberculosis* by PCR. Level of IL-2 receptor was increased to 15,000 U/mL (ref 45–1100), and NK-cell activity was slightly above the normal range at 64% (ref 18–59).

Taken together, the patient met seven out of eight diagnostic criteria for HLH as stated by the HLH-2004 trial (Table 1) [14]. Miliary TB was regarded as the triggering factor. The TB isolate was likely susceptible to rifampicin and isoniazid, the most important first-line anti-TB drugs, as line-probe assay (GenoType MTBDRplus, Hain Lifescience) showed no mutations conveying resistance to rifampicin or isoniazid in the *rpoB*, *katG* and *inhA* genes. Thus, the patient received standard anti-TB treatment with rifampicin, isoniazid, pyrazinamide and ethambutol, in the form of four-drug fixed-dose combination tablets (FDCs) [15].

Treatment of secondary HLH is directed against the underlying disease; however, initial treatment with steroids and/or immunosuppressants is usually required to prevent rapidly progressing organ damage and death. Etoposide was discontinued after 3 weeks due to increasing cytopenia, while steroids were continued with tapering doses every 14 days over a 6-week period (Fig. 1). Towards the end of the hospital stay the patient received 5 days of additional treatment with intravenously administered ceftriaxone, due to a febrile condition probably related to neutropenia (neutrophils at 0.1 10^9^/L). His general clinical condition was rapidly improving, and after 1 month of hospitalization he was discharged with biochemical recovery including decreasing serum ferritin. In light of the severe initial disease, the initial-phase anti-TB-treatment with four-drug FDCs was extended for a total of 3 months, and the subsequent continuation-phase treatment with two-drug FDCs containing rifampicin and isoniazid was given for 6 months, for a total treatment duration of 9 months. On subsequent outpatient visits, the patient’s condition has continuously improved, and he is currently without any sequela or signs of active disease.

## Discussion

HLH is a life-threatening immune syndrome caused by the massive production of cytokines due to a highly stimulated but ineffective immune response. The pathogenesis of HLH is complicated and not completely understood, but secondary hyperactivation of macrophages and CD8+ T lymphocytes in the absence of regulatory NK cell activity seems essential. This results in the massive production of proinflammatory cytokines that directly provokes end organ damage [1–6]. The diagnosis of HLH is challenging and can often be overlooked or delayed. As no single diagnostic test exists, the diagnostic criteria applied in the HLH-2004 trial are currently used to diagnose HLH (Table 1). Hyperferritinemia is often the first clue to the diagnosis. Although the diagnostic criteria consider ferritin > 500 µg/L as diagnostic, the ferritin levels are often considerably more elevated [16], as was the case for our patient presenting with serum ferritin increasing to > 17,000 µg/L. If additional cytopenias are present, involving at least two lines, this should clearly raise the clinical suspicion of HLH. Our patient developed thrombocytopenia and anemia, which led to further investigation for suspected HLH. Fasting hypertriglyceridemia and/or hypofibrinogenemia can also quite easily be detected, while assays for soluble IL-2 receptor and NK cell activity are often not readily available in standard hospital laboratories. A biopsy demonstrating hemophagocytosis is of considerable help, as in our patient (Fig. 2). Bone marrow biopsies are easily obtained, and mandatory to rule out secondary causes of HLH such as underlying malignant disorders or visceral leishmaniasis.

The etiology of HLH can be categorized as primary and secondary causes (Table 2). Primary HLH is very rare in adults and is caused by loss of function in genes associated with vesicle trafficking in cytotoxic T and/or NK cells [17]. This results in reduction or loss of the cytotoxic potential in NK and T cells, and inability of the immune system to sufficiently eliminate activated macrophages [18, 19]. Although primary HLH is caused by an underlying genetic immunodeficiency, clinical presentation is usually triggered by a viral infection, most commonly EBV [20]. Secondary HLH can be triggered by both malignant and nonmalignant diseases (Table 2) [21]. Malignant disorders often associated with secondary HLH include hematological malignancies such as lymphoma, acute leukemias and myelodysplastic syndrome, but the syndrome has also been observed in relation to solid tumors [22]. The nonmalignant conditions associated with HLH can be broadly divided into autoimmune disorders and infectious diseases. Among autoimmune disorders we find diseases such as systemic lupus erythematosus (SLE), systemic-onset juvenile idiopathic arthritis (Still’s disease) and rheumatoid arthritis. The most common infections reported to be associated with HLH include EBV, leishmaniasis, CMV, HIV and fungal infections [12], and most recently SARS-CoV-2 [23]. The association between TB and HLH has been described previously. However, most of the cases described are in immunocompromised patients, including patients with concomitant malignant disorders, HIV, patients on hemodialysis and renal transplant recipients [11].

**Table 2:** Classification of HLH

Primary HLH	Secondary HLH			
	Malignant		Nonmalignant	
	Hematological malignancies	Solid tumors	Autoimmune	Infectious
Genetic defects impairing NK and T cell function	Lymphoma Acute leukemias MDS	Lung GI tract Pancreas UG tract	SLE Still’s disease Rheumatoid arthritis	EBV Leishmania CMV SARS-CoV-2 HIV Protozoa Fungal infections TB

*MDS* myelodysplastic syndrome, *GI tract* gastrointestinal tract, *UG tract* urogenital tract *SLE* systemic lupus erythematosus, *EBV* Epstein–Barr virus, *CMV* cytomegalovirus, *HIV* human immunodeficiency virus, *TB* tuberculosis

Diagnosis of pulmonary TB is rapid and straightforward when acid-fast bacilli can be seen on direct microscopy. However, negative microscopy does not rule out TB. Extrapulmonary TB is particularly challenging to diagnose, as direct microscopy and PCR have low sensitivity on specimens such as pus, cerebrospinal fluid, biopsies and lymph nodes. Although TB bacteria are present throughout the lungs in miliary TB, the majority of these patients have no visible acid-fast bacilli on sputum microscopy. As in our case, definitive diagnosis of microscopy-negative TB relies on a culture for *M. tuberculosis*, which may take up to 6–8 weeks to become positive. While PCR has high specificity and can rapidly identify *M. tuberculosis*, its sensitivity is far inferior to culture.

The classical description of miliary TB is that of hematogenous seeding occurring successively after primary infection and resulting in millet-like grains in various organs, including the lungs, where these grains result in typical findings on chest X-ray. Pathological examination typically shows granulomas, as was found on the bone marrow specimen from our patient, and is highly suggestive of the diagnosis. The term cryptic miliary TB is sometimes used to describe similar seeding occurring later in conjunction with reactivated TB ("post-primary" TB), and tending to affect older individuals, often without typical X-ray findings. A third form, nonreactive TB, describes widespread seeding of TB where there is dysfunctional immune response, allowing the bacteria to spread without formation of granulomas, and frequently without “miliary” characteristics on chest X-ray. Nonreactive TB often occurs in immunocompromised patients, including HIV patients with low CD4 (cluster of differentiation 4) counts. The term disseminated TB is often used interchangeably with miliary TB, but sometimes reserved for nonreactive TB or widespread TB without typical miliary findings on chest X-ray. The patient's origin in a TB-endemic region suggested that he suffered from cryptic miliary TB due to reactivation many years after primary infection. Nonreactive TB was unlikely, as he had granuloma formation on pathological examination, and no underlying immunosuppression could be found. The National Reference Laboratory for Mycobacteria (NRL) at the Norwegian Institute of Public Health performed phenotypical drug susceptibility testing using the BACTEC MGIT 960 system (Becton Dickinson, NJ, USA), confirming susceptibility to all primary anti-TB drugs, as well as relatedness analysis with comparison to isolates from patients diagnosed with TB in Norway during the last 8 years. Interestingly, MIRU-VNTR (mycobacterial interspersed repetitive unit-variable number tandem repeat) typing first suggested that the isolate could be related to one obtained from a patient diagnosed with TB in Norway 3 years earlier, consistent with relatively recent primary infection causing classic miliary TB. However, whole-genome sequencing (on an Illumina platform) excluded relatedness to this isolate (in-house methodology), supporting the initial theory of cryptic miliary TB caused by reactivation decades after primary infection.

Diagnostics of TB-triggered HLH is particularly challenging, as HLH and miliary TB have several features in common, particularly fever, splenomegaly and anemia, but also lymphopenia, thrombocytopenia and elevated ferritin. While anemia is seen in most miliary TB patients, only up to one quarter have lymphopenia or thrombocytopenia as well [24]. Thrombocytopenia in TB can result from immune-mediated destruction, hypersplenism and infiltration of the bone marrow [25]. While ferritin is usually moderately increased in TB, values in excess of 10,000 ng/mL have been described in miliary TB [24]. Our patient’s symptoms and findings coincided with the multiple sites from which *M. tuberculosis* was recovered, but could also represent organ damage due to HLH. Hence pollakiuria and renal failure could be due to affection of kidneys and the urinary tract, history of diarrhea, colonic changes and abdominal fat tissue reaction on CT due to affection of the intestines, and notably, dyspnea, pulmonary crepitation, respiratory failure and ground-glass opacities on CT due to affection of the lungs. The bone marrow biopsy confirmed evidence of both hemophagocytosis and TB. The pathophysiology of HLH related to TB is mainly unknown. Phagocytosis of *M. tuberculosis* by macrophages, and hence Th1-mediated cytotoxicity, followed by release of a large quantity of cytokines and chemokines are probably involved [11]. HLH due to TB has a high mortality rate, and most reports describe fatal outcome, particularly if other complicating factors are present [11, 26, 27]. The treatment of HLH is challenging, and simultaneous immunosuppressive treatment for HLH combined with specific treatment of the underlying condition is necessary [16]. Given the rarity of the disease, few studies describing treatment alternatives exist. In most treatment regimens, etoposide is combined with corticosteroids [16, 28, 29].

The greatest obstacle to a successful outcome for individuals with HLH is a delayed diagnosis. As soon as the diagnosis is suspected or confirmed, treatment should be initiated. The major aims for HLH therapy are to suppress the life-threatening inflammation and to treat the underlying cause. Therapy based on the HLH-94 and HLH-2004 protocols consists of a series of weekly treatments with corticosteroids and etoposide, with the addition of intrathecal methotrexate and hydrocortisone for those with central nervous system (CNS) involvement (14, 30). For patients with an underlying infection, treatment of the triggering condition should be initiated simultaneously, as treatment of the trigger has the potential to remove the stimulus for immune activation. For the current case, the underlying condition, TB, was treated with rifampicin, isoniazid, pyrazinamide and ethambutol. However, in the case of TB-triggered HLH, it is particularly challenging that the immunosuppressive treatment indicated for HLH can severely exacerbate the course of TB. In the absence of tuberculostatic drugs, the treatment for HLH would have impaired the patient’s immunity to an extent that could result in fulminant, disseminated TB. Beyond this rare situation of HLH, miliary TB should always be considered before instituting immunosuppressive treatment for febrile illnesses of suspected immunological genesis. While treatment for TB was critical for the survival of the patient, therapy against HLH seemed appropriate as well, and in accordance with generally accepted treatment algorithms. This is supported by the fact that our patient had an improvement of his respiratory failure after treatment with steroids and anti-TB drugs was initiated, but only had improvement in his kidney and bone marrow failure after etoposide was added.

To conclude, TB-associated HLH is an exceedingly rare condition, but should be considered for patients with risk factors for TB presenting with severe signs of organ failure, and clinical or laboratory findings consistent with HLH. The incidence of TB is declining globally, but it is still highly endemic in some countries. Awareness of HLH as a complication of TB and other chronic infectious diseases such as HIV, leishmaniasis and hepatitis is important for early diagnosis and adequate management.

## Data Availability

Not applicable.

## Abbreviations

ANA: Antinuclear antibody
ANCA: Antineutrophil cytoplasmic antibody
CMV: Cytomegalovirus
CRP: C-reactive protein
CT: Computed tomography
EBV: Epstein–Barr virus
HIV: Human immunodeficiency virus
HLH: Hemophagocytic lymphohistiocytosis
PCR: Polymerase chain reaction
TB: Tuberculosis
WBC: White blood cell count
